# Synthesis, Anti-Inflammatory and Anti- Nociceptive Activities and Cytotoxic Effect of Novel Thiazolidin-4-ones Derivatives as Selective Cyclooxygenase (COX-2) Inhibitors

**Published:** 2013-12

**Authors:** Seyed Adel Moallem, Mohsen Imenshahidi, Narges Shahini, Ahmad Reza Javan, Mohsen Karimi, Mona Alibolandi, Morteza Ghandadi, Leila Etemad, Vahidehsadat Motamedshariaty, Toktam Hosseini, Farzin Hadizadeh

**Affiliations:** 1 Pharmaceutical Research Center, Mashhad University of Medical Sciences, Mashhad, Iran; 2 Medical Toxicology Research Center, Mashhad University of Medical Sciences, Mashhad, Iran; 3 Department of Pharmacodynamics and Toxicology, School of Pharmacy, Mashhad University of Medical Sciences, Mashhad, Iran; 4 Biotechnology Research Center, Mashhad University of Medical Sciences, Mashhad, Iran; 5 Department of Medicinal Chemistry, School of Pharmacy, Mashhad University of Medical Sciences, Mashhad, Iran

**Keywords:** Antinociceptive, Anti-Inflammatory, Anticancer, Celecoxib, COX-2 inhibitor, Thiazolidin-4-ones

## Abstract

***Objective(s): ***Nowadays, *COX-2 inhibitors* such as valdecoxib *are removed from the market* *because* of their *cardiovascular* toxicity and their potential to increase the risk of strokes. In response to this, medicinal chemists have attempted to synthesize new classes of COX-2 Inhibitors.

***Materials and Methods:*** In this study, three novel analogues of thiazolidin-4-ones derivatives **2a-c** were synthesized. The ability of these compounds to inhibit ovine COX-1 and COX-2 (0.2- 0.8 µM) was determined using a colorimetric method. The cytotoxic effect of the synthesized compounds (25-100 M) was also investigated by measuring their cytotoxicity against Caco-2 and MCF-7 cell lines using MTT assay. Cell apoptosis was determined by ﬂow cytometry. Writhing test (7.5-75 mg/kg) was used to examine the antinociceptive effects in mice. The effect of the analogues against acute inflammation (7.5-75 mg/kg) was also studied using xylene-induced ear edema test in mice.

***Results: ***The synthesized compounds showed a weak capacity to inhibit the proliferation of Caco-2 and MCF-7 cell lines. The COX-2 inhibition potency and selectivity index for test compounds **2a–b** were as follows; celecoxib > **2b** > **2a**. On the other hand, all three analogues exhibited strong antinociceptive activity against acetic acid-induced writhing. The anti-inflammatory and antinociceptive effects of the analogues were markedly more than positive control, celecoxib.

Conclusion: This study demonstrates that the antinociceptive and anti-inflammatory activity profiles exhibited by the novel synthesized compounds are independent from their COX-2 inhibitory potencies. The found antinociceptive and anti-inflammatory effects can be caused by interaction with other target; independent from COX-2. Accordingly, the compounds 2a-c could serve as lead compounds to develop novel anti-inflammation and antinociceptive drugs.

## Introduction

Cyclooxygenase participates in the biosynthesis of prostaglandins, biologically active chemicals that are involved in numerous physiological processes but also in pathological conditions such as inflammation. Cyclooxygenase exists under two isoforms: a constitutive (COX-1) and an inducible form (COX-2) (1). Both enzymes are inhibited by non-steroidal anti-inflammatory drugs (NSAIDs). The COX-1 isozyme which is involved in various homeostatic processes has a housekeeping role. 

 COX-1 is also involved in the synthesis of cytoprotective prostaglandins in the gastrointestinal tract and the pro-aggregatory thromboxane in blood platelets while COX-2 expression is associated with inflammation and cancer (2-4).

Classical NSAIDs as non-selective COX inhibitors have been widely used in the treatment of several chronic illnesses for a long time. Having anti- inflammatory, analgesic and antipyretic properties, this class of agents is mainly used to treat chronic inflammation conditions. However, all those agents cause problematic side-effects related to COX-1 inhibition of which gastrointestinal irritation (5, 6), occasionally leading to hemorrhage and ulceration, is the most typical one. Later, the confirmation of therapeutic advantage arising from selective COX-2 inhibition led to the development of COX-2 selective inhibitors. However, recent studies reveal that the long term use of selective COX-2 inhibitors is limited because of cardiovascular thrombotic events related to the aggregatory properties of these drugs (7). COX-2 mediates the biosynthesis of prostacyclin in vascular endothelium. Prostacyclin has important physiological roles, such as increasing blood flow to injured tissues, reducing leukocyte adherence, and inhibiting platelet aggregation, thereafter, the inhibition of its production by selective COX-2 inhibitors might result in adverse effects on the cardiovascular system (8, 9). 

**Figure 1:**
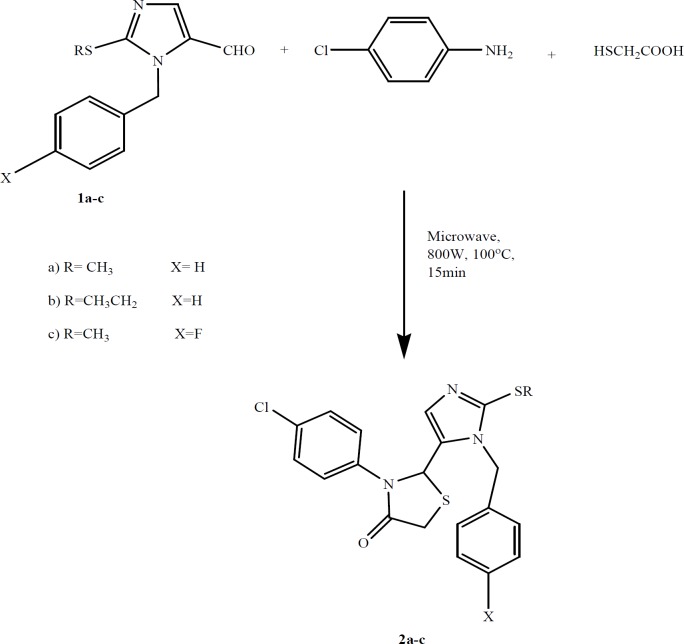
**Schematic representation of chemical** **synthesis** **of Thiazolidin-4-ones derivatives 2a–c**

In this study, compounds with moderate selectivity for COX-2 were synthesized to reduce possible cardiovascular side effects. Here, we report the synthesis of a new series of thiazolidin-4-ones derivatives as a novel class of NSAIDs. Also, their anti-inflammatory, antinociceptive, cytotoxic activity and structure-activity relationships were investigated. 

## Materials and Methods

***General***

All reagents and solvents were obtained from commercially available suppliers (Merck and Sigma Aldrich) and used without further purification except for toluene which was dried over sodium, prior to use. Melting points were recorded on Melting Point Electrothermal apparatus (England) and are uncorrected. ^1^H NMR spectra were recorded on a Bruker Ac-80 (Germany) spectrophotometer with all chemical shifts reported in ppm relative to internal tetramethylsilane (TMS). 

Elemental Analyzer Costech ECS 4010 (Italy) was used and the C, H, N results were within ±0.4% of the theoretical values. 

***Chemical methods***

The desired compounds were synthesized by reactions outlined in Figure 1. Imidazole aldehydes (**1a-c**) were prepared as described previously (10). Then, the resultant aldehydes **1a-c** were reacted with 4-chloroaniline in the presence of thioglycolic to obtain thiazolidin-4-ones derivatives **2a-c**. 

***General*** ***procedure*** ***for*** ***synthesis*** ***of*** ***2-(1-benzyl-2-alkylthio-5-imidazolyl)-3-(4-chlorophenyl) - 1,3-thiazolidin-4-ones (2a-c) ***

The mixture of **1a-c**, 4-chloroaniline (8 mmol) and thioglycolic acid (16 mmol) in dry toluene (50 ml) was added to a glassy flask fitted with a condenser and was placed in a Milestone synthesis Microwave apparatus (Italy). The mixture was irradiated and stirred at 800 W while temperature was kept at 100°C for 15 min. The reaction mixture was extracted by addition of saturated sodium bicarbonate (3x, 50 ml) and the organic phase was separated. After removing toluene under vacuum, residues were dissolved in 50 ml methanol. Sufficient amount of water was added to the resultant methanolic solution to give a milky white precipitate of **2a-c**.

***2-(1-benzyl-2-methylthio-5-imidazolyl)-3-(4-chloro-phenyl)-1,3-thiazolidin-4-ones*** *(* ***2a*** *)*

This compound was obtained with 30% yield; mp: 95-96°C; ^1^H-NMR (CDCl_3_): δ 2.6 (s, 3H, CH_3_), 4.7(m, 2H, CH_2_ thiazolidone), 5.8 (s, 2H, CH_2_N), 6.7-7.5 (m, 9H, arom), 7.6 (s, 1H, H-C_4_ imidazole), 8.3 (s, 1H, H-C_2 _thiazolidine). *Anal*. Calcd. for C_20_H_18_ClN_3_OS_2_ : C, 57.75; H, 4.36; N, 10.10. Found: C, 57.79; H, 4.35; N, 10.12.

***2-(1-benzyl-2-ethylthio-5-imidazolyl)- 3-(4-chloro-phenyl)-1, 3-thiazolidin-4-one (2b)***

This compound was obtained with 46% yield; mp: 97-98 ⁰C; ^1^H-NMR (DMSO-d_6_): δ 1.3 (t, 3H, CH_3_), 3.2 (q, 2H, CH_2_S), 4.7(m, 2H, , CH_2_ thiazolidone), 5.8 (s, 2H, CH_2_N), 6.7-7.5 (m, 9H, arom), 7.6 (s, 1H, H-C_4_ imidazole), 8.3 (s, 1H, H-C_2 _thiazolidine).* Anal*. Calcd. for C_21_H_20_ClN_3_OS_2_ : C, 58.66; H, 4.69; N, 9.77. Found: C, 58.45; H, 4.71; N, 9.79.

***2-[1-(4-fluorobenzyl)-2-methylthio-5-imidazolyl]-3-(4-chlorophenyl)-1,3-thiazolidin-4-ones (2c)***

This compound was obtained with 42% yield; mp: 94-95°C; ^1^H-NMR (CDCl_3_): δ 2.6 (s, 3H, CH_3_), 4.7(m, 2H, CH_2_ thiazolidone), 5.8(s, 2H, CH_2_N), 6.7-7.5 (m, 8H, arom), 7.6 (s, 1H, H-C_4_ imidazole), 8.3 (s, 1H, H-C_2 _thiazolidine).* Anal*. Calcd. for C_20_H_17_ClFN_3_OS_2_: C, 55.36; H, 3.95; N, 9.68. Found: C, 55.18; H, 3.96; N, 9.64.

***Cell culture***

Human tumor cell lines of two different histological origins were used: Caco-2 (human colorectal cancer) and MCF-7 (human breast cancer) which were purchased from the National Cell bank of Pasteur Institute of Iran, Tehran, Iran.

Stock cell cultures were grown in RPMI 1640 medium (Gibco, USA) supplemented with 10% fetal bovine serum (FBS) and 100 unit per ml of penicillin-streptomycin (Sigma, USA) at 37°C in humidified incubator with 5% CO_2_ atmosphere. After three day incubation, the cells were detached using 0.25% Trypsin-0.05% EDTA solution (Biogene, Iran), and resuspended in RPMI 1640 medium containing 10% FBS. Cell count and viability was determined by Trypan blue staining followed by hemocytometry.

***Colorimetric method for quantification of cytotoxicity (MTT assay)***

Cells distributed in 96-well plates (2×10^4^ cells/well) were exposed to various concentrations of synthesized analogs **2a-c** or celecoxib (25 M, 50 M, 75 M and 100 M) at 37°C, 5% of CO_2_ in air for 24, 48 and 72 hr. After incubation periods, 3-(4,5-Dimethylthiazol-2-yl)-2,5-diphenyltetrazolium bromide , MTT (Sigma, USA) was added to each well at the concentration of 0.5 mg/ml. Subsequently, microtiter plate was incubated for an additional 3 hr at 37°C and each medium was then removed prior to the addition of dimethyl sulfoxide to dissolve the formazan crystal formed by viable cells. Absorbance was measured at 545 nm using a microplate reader and demonstrated as a percent relative to untreated control cells.

***In vitro*** **COX inhibition assay**

The potency of the synthesized compounds and celecoxib to inhibit ovine COX-1 and COX-2 (0.2, 0.3, 0.4, 0.6, 0.8 µM) was measured using enzyme-immuno assay (EIA) kit (Cayman Chemical Company, Item Number 760111, USA) according to the manufacturer instructions. The same kit for determination of COX-1 and COX-2 inhibition was used by us previously (11).

***Apoptosis assay***

Caco-2 or MCF-7 cells (1×10^6^ cells/ml) were exposed to IC_50_ concentration of compounds **2a-c** or celecoxib to induce apoptosis. A suspension of untreated Caco-2 or MCF-7cells (1×10^6^ cells/ml) was used as negative control. Both control and test samples of cell cultures were incubated for 24 hr at 37°C and 5% CO_2_ atmosphere. After 24 hr incubation, adherent cells were collected, washed three times with phosphate-citrate buffer (0.2 M, pH 7.8) and fixed for 24 hr with cool ethanol at 4°C. Propidium iodide was then added to a final concentration of 50 µg/ml for DNA staining and the cells were incubated and light protected at room temperature for 3 hr. The fluorescence of the cells was analyzed in a FACScan flow cytometer (Becton Dickinson, USA). The obtained cytometric data were analyzed using WinMDI 2.8 software (The Scripps Research Institute, USA).

***Animals***

Animal experiments were carried out using 20-30 g male BALB/c mice purchased from Razi Institute, Mashhad, Iran. Animals were kept in plastic cages at room temperature of 21±2°C and 12 hr light/dark cycle. All experiments were performed according to the National Institute of Health guidelines for the care and use of laboratory animals and to the European Community Directive for the Care and use of Laboratory Animals of 1986 (86/609/EEC), and approved by our Institution’s Animal Care Committee. 

***The maximum non-fatal doses***

Various doses of the synthesized compounds were dissolved in a solution of NaCl 0.90% w/v, containing carboxymethylcellulose sodium (*CMC*) *0.5*% w/v and administered to groups of six mice via intraperitoneal injection (IP). The number of dead mice was counted during 48 hr after injection.

***Writhing test***

The nociceptive responses in mice were investigated using writhing test. One hr after administration of different concentrations of the compounds (7.5, 30, 52.5 and 75 mg/kg) and celecoxib (75 mg/kg) to male BALB/c mice, each mouse received one IP injection of acetic acid solution 0.7% (v/v) (0.1 ml/10 g). The number of abdominal constrictions (writhing episode) seen in treated animals was counted during a 30 min period (12). Celecoxib and normal saline was respectively used as positive and negative control.

***Xylene-induced ear edema***

The effect of the analogues against acute inflammation was studied using xylene-induced ear edema test in mice. 

Animals were treated via IP injection of the compounds (7.5, 30, 52.5, 75 mg/kg) and celecoxib (75 mg/kg).

Thirty minutes later, edema was induced in each group by applying 0.03 ml of xylene to the inner surface of the right ear. After 2 hr, the mice were sacrificed under ether anesthesia and both ears were removed, sized and weighed. The increase in weight caused by the irritant was measured by subtracting the weight of the untreated left ear section from that of the treated right ear sections (12).

***Modeling and docking studies***

 Because there is not any crystal structure for human COX-2 protein, by similarity search of human COX-2 protein against UniProt database (www. Uniport.org), mouse COX-2 protein (UniProt ID: Q05769) was selected based on the quite high identity, 86%, instead of human COX-2 protein for docking investigations. The crystal structure of protein was downloaded from protein data bank (PDB ID: 1cx2) and further preparations like removing water molecules and adding polar hydrogens were done using AutoDock tools software. Ligands were drawn by ChemDraw 8 software and 3D structures were prepared using molecular mechanic force field and semi-empirical AM1 calculations in Hyperchem 7. The energy minimized ligands were docked in the PDB file 1cx2 after SC-558 (the inhibitor which co-crystallized in active site of enzyme) was deleted. Docking studies were performed using AutoDock software Version 4.2. Automated docking simulation was applied to dock **2a-c **into the active site of murine COX-2 enzyme using Lamarckian genetic algorithm. The regions of interest of the enzyme were defined as a box size of 23.947, 21.582 and 15.436 angstrom in X, Y and Z axis. Important pdbqs, gpf and dpf files of both protein and ligands were produced by AutoDock tools while further docking was performed using related commands. Accuracy of docking protocol was examined by docking SC-558 (co-crystallized inhibitor)between interested parts of protein. The program generated docked conformers corresponding to the lowest-energy structures. After docking procedure, docking results were submitted to be visualized for further evaluations. Docking analysis and picture preparation was done using MOE software.

**Table 1:** *In vitro* COX-1 and COX-2 enzyme inhibition data

Compound	IC50 (µM) a		Selectivity index b
	COX-1 COX-2		
Celecoxib	5.07	0.13	39
2a	15.59	0.82	18.82
2b	16.70	0.71	23.42

a Values are mean values of two determinations acquired using an ovine COX-1/COX-2 assay kit

b *In vitro* COX-2 selectivity index (COX-1 IC50/COX-2 IC50)

***Statistical analysis***

The values were expressed as ED_50_ or IC_50_ of the compounds.CalcuSyn version 2 (www.biosoft.com) was used for calculations. Data were analyzed by one way analysis of variance (ANOVA) followed by the multiple comparison test of Tukey–Kramer.

**Table 2:** The anti-proliferative activities for compounds **2a-b** and celecoxib

Compound	IC50 (µM)^a^	
	Caco-2 MCF-7	
Celecoxib	50.13	76.31
2a	106	121
2b	82.28	109.92

a Values are means of three experiments, where the deviation from the mean is <10% of the mean value

**Table 3:** Values of ED_50_ of compounds in writhing test

Compound	ED_50_(mg/kg)
2a	15.112
2b	39.873
2c	8.7636
celecoxib	ND*

* Celecoxib at single dose of 75mg/kg decreased number of writhing by 57.5% relative to control

## Results

***In vitro cyclooxygenase (COX) inhibition***

The alkylthio substituent on the imidazole ring of **2a** and **2b** was changed to determine the effects of alkyl chain length substituent on COX-1 and COX-2 inhibitory potency and selectivity. The COX-2 inhibition potency for test compounds **2a–c** was celecoxib >**2b** > **2a** and results for selectivity index were celecoxib > **2b** > **2a** (Table 1). Synthesized compounds exhibited weak inhibition of both COX-1 and COX-2 compared to celecoxib. On the other hand, obtained results demonstrated that by changing the alkyl chain length of the substituent at position 2 in the imidazole ring from methylthio to ethylthio, compound **2b** showed little increase of selectivity and cyclooxygenase inhibition potency.

***Growth inhibition assay***

The synthesized thiazolidin-4-ones derivatives **2a-c** were investigated for anticancer activity *in vitro* in Caco-2 and MCF-7 cancer cell lines by the MTT assay with celecoxib as positive control. In comparison with celecoxib, compounds **2a** and **2b** showed no considerabele activity against Caco-2 and MCF-7 cell lines. On the other hand, our results on biological activity indicated that the introduction of ethylthio on the imidazole ring of synthesized compounds is effective as, **2b** showed more activity than the analogue **2a**, which has a methylthio on imidazole ring (Table 2). Maximal effects of synthesized compounds were observed at 24 hr after treatment (Data for 48 and 72 hr not shown).

***Cell cycle analysis***

**Figure 2:**
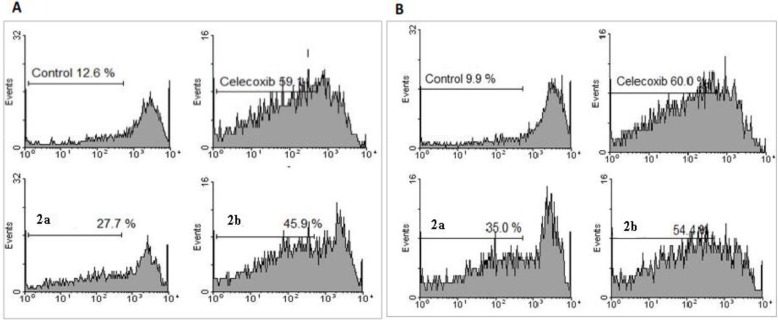
**Effects of compounds 2a–b and celecoxib on apoptosis induction were** **quantified** **as percent sub-diploid cells. A) MCF-7 cells. Untreated MCF-7 cell was used as control; B) Caco-2 cells. Untreated Caco-2 cell was used as control. The results of flow cytometry was not determined for compound 2c**

**Figure 3:**
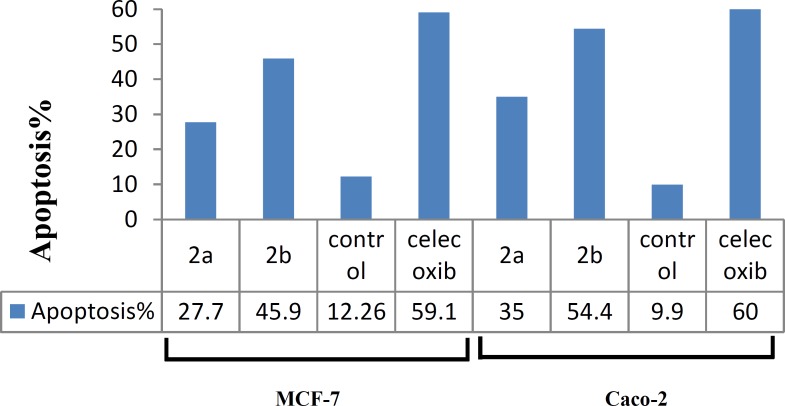
**Impacts of compounds 2a–b and celecoxib on apoptosis induction**

For determination of cells undergoing apoptosis, flow cytometry was performed. One of the classic methods is to monitor the subdiploid peak due to the loss of DNA small fragments using propidium iodide staining. As shown in Figure 2, the sub-G1 cell populations were observed clearly for all synthesized compounds as follows: celecoxib>**2b**>**2a**. Data of flow cytometry (Figure 3) are in agreement with MTT and COX-2 selectivity data.

***Pharmacological results***

The maximum 48 hr nonfatal doses of synthesized compounds were 75 mg/kg. Injection of four doses of analogues (7.5, 30, 52.5 and 75 mg/kg) and celecoxib (75 mg/kg) as positive control to mice showed potent anti- nociceptive effect in the writhing test. Anti-nociceptive effects of the new analogues (75 mg/ kg) were significantly more than celecoxib (75 mg/kg). ED_50_ values of the compounds and the positive control in writhing test are shown in Table 3.

**Table 4:** Values of ED_50_ of compounds in xylene induced ear edema test

Compound	ED_50 _(mg/kg)
2a	68.35
2b	73.59
2c	64
celecoxib	ND*

In xylene-induced ear edema test, all three analogues exhibited anti-inflammatory activity. Comparing to positive controls, the anti-inflammatory effect of compounds **2a-c** were meaningfully more than the positive control, celecoxib (75 mg/kg). Table 4 represents values of ED_50_ for compounds **2a-c** and celecoxib in xylene induced ear edema test.

***Docking***

**Figure 4:**
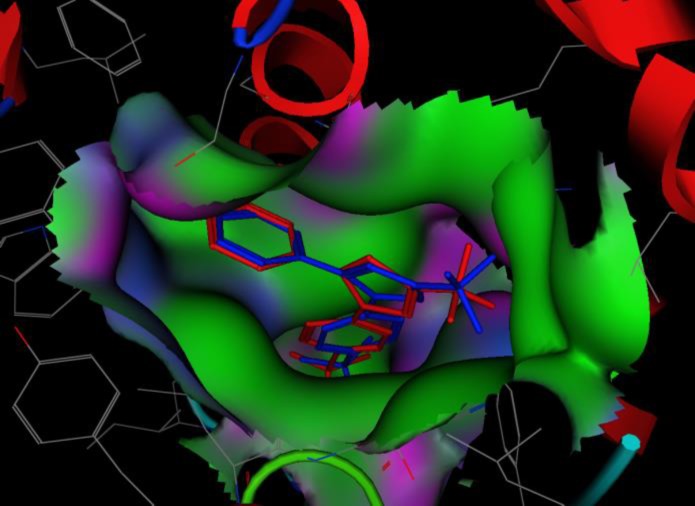
Docked SC-558 (blue) and co-crystalized one (red) are almost at the same position revealing the accuracy of docking protocol

**Figure 5:**
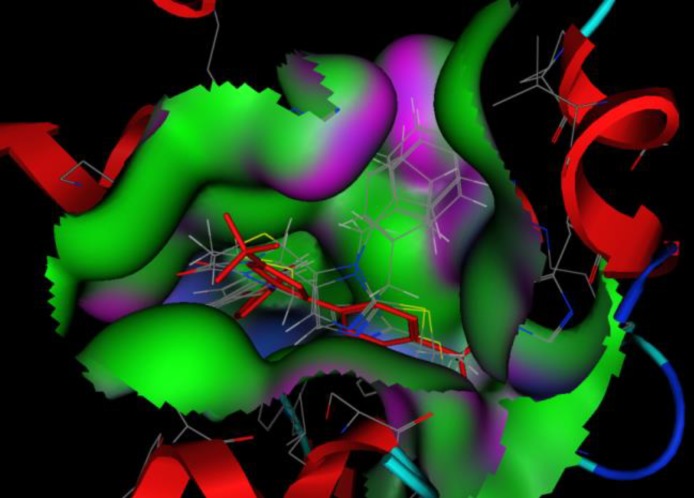
Docked compounds in active site cavity of enzyme, crystalized SC-558 is shown in red stick lines

**Figure 6:**
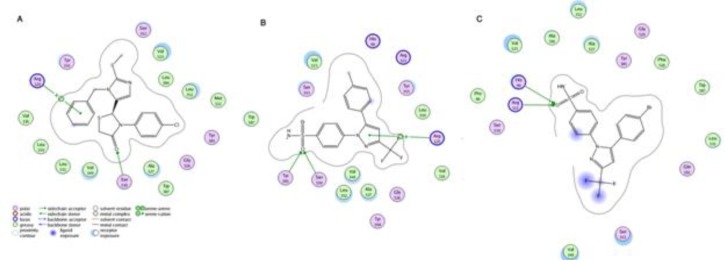
**Interactions between 2a (A), celecoxib(B), SC-558 (C) and mouse COX-2 enzyme**

Figure 4 shows docked SC-558 at the same position as co-crystallized one in active site of enzyme (RMSD=0.641), revealing that docking protocol was reasonable. Docking results illustrated that all compounds are in appropriate position among the active site of murine COX-2 (Figure 5). Hydrogen bond between carbonyl group and Ser530 residue and an arene-cation interaction at aromatic ring was formed in all compounds (Figure 6) but celecoxib mostly interacts with murine COX-2 through hydrogen bond between Tyr385 and Ser530 with the carbonyl group and an arene-cation interaction with Arg120 and SC-558 just have shown hydrogen bond interactions. It is worthy to mention that site-directed mutation study have demonstrated that Tyr385 and Ser530 are crucial residues for enzyme action and they have roles in interaction between inhibitors and enzyme, and our docking results are in agreement with Rowlinson *et al* findings (13). 

**Table 5:** *In silico *COX-2 enzyme inhibition data from AutoDock

compound	K_i _(nM)	Binding energy(kcal)
2a	827.65	-8.3
2b	645.75	-8.44
2c	384.2	-8.75
celecoxib	14.13	-10.71

As a result, it was proved that hydrogen bonds formation between ligand and murine COX-2 are crucial in anti-proliferative and COX-2 selectivity effects. Binding energies and inhibition constant values (Ki) are summarized in Table 5. 

## Discussion

Here we have described a straightforward and efficient synthesis of new strong anti-inflammatory and anti- nociceptive compounds containing 1-benzyl-2-alkylthio-5-imidazolyl and 4-chlorophenyl at the 2 and 3 position of thiazolin-4-ones pharmacophoric group. 

The cytotoxic activity of the synthesized compounds was weak and comparable to that of celecoxib. Cell cycle analysis approved that apoptosis mechanism is probable. In previous studies, clinical trials have revealed that a dose of 400 mg celecoxib twice daily decreased polyp growth while this effect was insufficient at 100 mg twice daily and rofecoxib does not exhibit cytotoxic activity even at relatively high doses (14, 15). Also our previous study showed that there was a relationship between COX-2 inhibition selectivity index and cytotoxic activity of COX inhibitors (16).

Synthesized compounds exhibited moderate selectivity for COX-2, less than that of celecoxib. Molecular modeling studies may explain these effects. In contrast to celecoxib less hydrogen binding was involved in ligand-protein interaction and it may be the reason for less selectivity of our compounds in comparison to celecoxib.

Although selective COX-2 inhibitors had a markedly lower rates of gastrointestinal adverse effects, increasing selectivity also increases the cardiovascular side effects by tipping the balance of prostacyclin and thromboxane (a pre-thrombotic eicosanoid) toward vasoconstriction and *thrombosis* (17, 18). Therefore, synthesized compounds **2a-c** with moderate selectivity may have less cardiovascular side effects.

Compound **2c** had the strongest *in vivo* anti-inflammatory activity which exceeds that of the parent reference, celecoxib, followed by **2a** which showed more activity than celecoxib. Compound **2c **also showed greater activity regarding anti-nociceptive effect, followed by** 2a** which exhibited more activity than celecoxib. The antinociceptive and anti-inflammatory activity profiles exhibited by the novel synthesized compounds were independent of their COX-2 inhibitory potencies. The found antinociceptive and anti-inflammatory effects can be caused by interaction with other target; independent from COX-2.

## Conclusion

 This study demonstrates that the synthesized compounds did not have significant anti-proliferative activities but exhibited antinociceptive and anti-inflammatory activity profiles, not related to their COX-2 inhibitory potencies. They could serve as lead compounds to develop novel anti-inflammatory and antinociceptive drugs.
